# Effect of component quality on sensory characteristics of a fish soup

**DOI:** 10.1002/fsn3.661

**Published:** 2018-05-08

**Authors:** Tommi E. M. Kumpulainen, Mari A. Sandell, Anu I. Hopia

**Affiliations:** ^1^ Functional Foods Forum University of Turku Seinäjoki Finland; ^2^ Functional Foods Forum University of Turku Turku Finland

**Keywords:** CATA, consumer study, food quality, freshness, modularity, sensory evaluation

## Abstract

The foodservice industry is a highly competitive branch where customer satisfaction and loyalty is dependent on the price and the quality of the food. To improve cost competitiveness, instead of fresh ingredients, more preprocessed items are used as components in dishes. This may impair the perceived product quality, and thus potentially decrease customer satisfaction. The effects of the component quality on a single dish were tested by serving fish soup in a consumer study (*n* = 205), and by serving the dish to an in‐house panel (*n* = 17) using a modified check‐all‐that‐apply method. The variable used for the quality of the fish and vegetable components was a previously unprocessed/fresh component being compared to a processed. This study showed that in a modular dish, each component had an effect on the perceived quality of the dish. When replacing a preprocessed component with a fresh one, the perceived pleasantness increased to a higher level. The fish as the main dish component had the largest effect on the quality. Fresh fish has the ability to enhance the taste of soup, even with frozen vegetables. The results from this study indicate that the effect of freshness can also be perceived in the cooked product.

## 1 INTRODUCTION

Foodservices are responsible for an emerging share of daily energy intake (Poti & Popkin, 2011; Lachat et al., 2012; Seguin, Aggarwal, Vermeylen, & Drewnowski, 2016). Lunch and other professionally prepared dishes served by restaurants, office canteens, and institutional cafeterias are important, but a highly competitive part of the foodservice sector. To maintain competitiveness, foodservice restaurants need to be able to serve good quality and reasonably priced dishes to customers. When processes are planned from the perspective of operational efficiency, there is a tendency for the sensory quality of food to be impaired (Creed, 2001). To be able to streamline the process and avoid an increase in labor costs, more preprocessed components are used (Rodgers, 2005; Rodgers & Assaf, 2006). Preprocessed food components require less work during preparation and produce less or no waste; thus, the total cost can be cheaper compared to fresh product (Puckett 2012a, 2012b). With this type of operational practice, the amount of fresh ingredients being used is now in the minority or even nonexistent in complete dishes (Engelund, Lassen, & Mikkelsen, 2007). Using raw materials with less than optimal quality may have an effect on the perceived quality of meals (Kumpulainen, Sandell, Junell, & Hopia, 2016; Swainson & McWatt, 2010). When the experienced quality is lower than expected, customer satisfaction and willingness to pay are typically decreased (Homburg, Koschate, & Hoyer, 2005; Liu & Jang, 2009; Seppä, Latvala, Akaichi, Gil, & Tuorila, 2015). The quality of dishes needs to be developed without adding cost or laborious steps into the production processes.

When using such elusive term as freshness, the term has to be determined in the current context. Freshness is a complicated term which, in relation to food, usually refers to recently harvested or unprocessed products (Cardello & Schutz, 2003). It is the sum of different sensory properties and can also be interpreted as a product possessing several ideal product characteristics (Péneau, Brockhoff, Escher, & Nuessli, 2007). In this case, freshness is used as an equivalent of unprocessed/nonfrozen and the opposite of nonfresh meaning preprocessed/frozen (Bremner, 2002); however, freshness has a wide variety of meanings and, for example, there is no universal definition in the case of fish (Bremner & Sakaguchi, 2000). A similar type of definition is used by the U.S Food and Drugs Administration: “The term “fresh,” when used on the label or in labeling of a food in a manner that suggests or implies that the food is unprocessed, means that the food is in its raw state and has not been frozen or subjected to any form of thermal processing or any other form of preservation” (U.S. National Archives and Records Administration). Typically, freshness is seen as a very important product property and it can have a significant effect on customer satisfaction (Cardello & Schutz, 2003; Ouellet & Norback, 1993). Machín, Giménez, Vidal, and Ares (2014) found that freshness is one of the main determinants of consumer choice in the purchasing context. The downside is that using fresh ingredients will possibly add to the cost of the production processes (Rodgers, 2005).

When cooking protein components or vegetables, they undergo some changes and seem to lose their fresh‐like properties (Cardello & Schutz, 2003). With regard to ingredients, which are not previously processed, the improved quality is easier to verify than with prepared or cooked raw materials (Borgogno, Favotto, Corazzin, Cardello, & Piasentier, 2015; Péneau, Linke, Escher, & Nuessli, 2009). It can be hypothesized that some of these fresh‐like attributes are still noticeable in the cooked products. Studies by Aaslyng and Frøst (2008, 2010) showed that as a part of a meal, the meat component interacts with the vegetables, and a liking of any individual components cannot predict very well an overall liking. Presumably, the meal components have some properties that make certain combinations more appealing than others (Nestrud, Ennis, Fayle, Ennis, & Lawless, 2011). In this study, two varieties of the same component were used, fresh as opposed to a preprocessed component. The dish components are considered as modules, where the quality of these modules can be varied depending on the end quality desired.

Mass customization (MC) is a production approach aimed at combining large volumes of mass production with the high quality of the individualized products (Pine, 1993). In an ideal situation, a company has a wide product variety manufactured with low unit costs. Modularity is one method of applying MC in practice (Parker, 2010). It is typically described as products broken down into separate components or modules, which can be reconfigured into multiple combinations (Tseng & Hu, 2014). This requires components to be compatible in order to compile a complete product or in this case a dish. Due to operational and economic efficiency, it is often applied in the manufacturing of strictly mechanical products (Fogliatto, Da Silveira, & Borenstein, 2012). The applicability of this method in food production is slightly more challenging due to complex interactions such as chemical and microbiological changes during processing (McIntosh, Matthews, Mullineux, & Medland, 2010; Swainson & McWatt, 2010). A study by (Adler‐Nissen et al., 2013). was conducted on the modularization of meal elements in the foodservice industry from the convenience and supply chain point‐of‐view. The results indicated that with new type of procedures, it is possible to accomplish prolonged shelf life and increased flexibility throughout the supply chain (Adler‐Nissen et al., 2013). Olsen and Aaslyng (2007) introduced the so‐called meal composition approach where modularization could be utilized as a tool by concentrating on the food quality. By combining these two approaches (Adler‐Nissen et al., 2013; Olsen & Aaslyng, 2007), the same type of model could also be used with a different emphasis. When optimizing the dish quality by partly replacing previously processed components with unprocessed, it should be possible to extract the best qualities for different combinations without compromising operational efficiency. Typically, modularity is used to enable flexibility by offering customers a wide variety of combinations. In this case, modularity is a tool which can be used to optimize the dish quality.

The objective in this study was to investigate whether a single food component and different component combinations would induce an effect on the sensory characteristics and consumer quality of a dish. The variable used for the component quality was a previously unprocessed component being compared to a processed component. The selected dish was fish soup with varying fish and vegetable mix components. For the test purposes, the component variable referred to in the study was the level of freshness. A secondary aim was to study whether the modular design could be applied to meal solutions and the dish quality increased without significantly increasing the raw material or labor costs. The effects of different components on the quality of the fish soup were tested with both a modified check‐all‐that‐apply method and a consumer study. To the authors’ knowledge, no study similar to this has been carried out with an actual commercial product.

## 2 EXPERIMENTAL

### 2.1 Description of fish soup samples

Altogether, four different fish soup samples were tested (Table 1). The fish soup was prepared using the following: water, stock, potato, fish, a mix of vegetables (carrots, onions, and leeks), and spices (salt, pepper, bay leaves, and dill). The standard product sources, methods, and recipes were used for the production of the soups. The same product recipe and raw materials are used in all the restaurants of the particular foodservice company selected. The freshness of the fish and vegetable mix components were varied in the different test samples. The fish components used in this study were rainbow trout (*Oncorhynchus mykiss*), both fresh and frozen. The frozen fish was a previously processed ready‐to‐use product cut into cubes. The fresh fish in this case was a previously unprocessed product, which was kept in ice, but was not frozen at any time during the supply chain. The fresh fish was delivered the same day it was used as a fillet, and manually cut into bite‐sized cubes. The vegetable mix component was also either fresh or nonfresh. The fresh vegetable components were delivered as complete, previously unprocessed vegetables and they were prepared (washed, peeled, and cut) just before cooking. The nonfresh components were frozen, minimally processed (washed, peeled, cut, and blanched), ready‐to‐use products. The quality of the potato and stock components was not varied between the different samples. The soup was made by cooking the potato and vegetable mix components in the stock. When the other components were cooked, the fish was added immediately before serving in order to minimize the holding time. All the test samples were prepared by experienced kitchen professionals. The kitchen personnel were aware that the study was conducted during the test days and that the raw material mix was modified, but the overall purpose of the study was not disclosed. To reduce the quality variance, the company uses the same standard recipe with exact amounts of each raw material by weight in order to minimize any personnel induced variance.

**Table 1:** Description of samples

Soup	Description
1	Fresh fish, fresh vegetables
2	Fresh fish, frozen vegetables
3	Frozen fish, fresh vegetables
4	Frozen fish, frozen vegetables

### 2.2 Sensory characteristics

The sensory study was carried out in two steps. In the first step, a 2‐hr session was conducted in the analytical sensory laboratory at the University of Turku (Functional Foods Forum). The sensory laboratory was designed according to the ISO 8589 standard. All the assessors (*n* = 8, females) participating in the session had more than 10 years’ experience in quantitative sensory profiling and descriptive methods. The samples used during the session were various commercially available fish soups from a Finnish food store. The samples were served cooked and warm. The purpose of the first step was to create a list of attributes of the different kinds of sensory properties of the fish soup samples, as regards flavor (including taste), smell, structure, and appearance. All the perceived properties were collected, listed, and discussed together before selecting the final list of descriptors/attributes. The attributes were grouped according to the sensory modality into four categories (odor, appearance, structure, and flavor).

In the second step, the four fish soup samples (Table 1) were evaluated by a sensory panel, which consisted of employees of a major foodservice company. Altogether, 17 panelists took part in the study. The study was conducted in two separate sessions, eight participants in the first session, and nine in the second. The company selected was a Finnish‐based company operating mainly in the Scandinavian market with a focus on foodservices, catering, and cafeterias. The recruited panelists, invited to this study on fish soup, were experienced in different in‐house testing methods that focus on products and concepts. Each participant evaluated all the four samples, one at a time. They were asked not to drink coffee or eat for an hour before the evaluation. The only preinformation given in relation to the sample was that the dish in question would be fish soup.

To discriminate the differences between the samples, a modified check‐all‐that‐apply (CATA) method was used (Ares, Tárrega, Izquierdo, & Jaeger, 2014). In the modified CATA method, the panelists were provided with a list of attributes created in the steps described above. The panelists were instructed to select all the descriptors that applied to the product in question. In the case of this study, there were altogether 72 descriptors, which were grouped according to the sensory modality into four categories (number of descriptors for each modality): odor (19), appearance (18), structure (17), and flavor (18).

Sensory evaluation was carried out in a silent room at ten separate test booths positioned around circular table. The warm soups were served to each participant individually by the kitchen personnel. All the samples were coded with a three‐digit number and served in a randomized order. Panelists were instructed to use all the senses to evaluate the soups and to check all the different descriptors that were appropriate to the soup in question. They were also instructed to rinse their mouth with water between samples. After finishing an evaluation, the panelist signaled to the staff to bring the next soup.

### 2.3 Consumer study

A total of 205 questionnaires were collected during three separate test days. The number of consumers for each soup sample was as follows: soup sample number 2 (fresh fish, frozen vegetables) 71 consumers, sample number 3 (frozen fish, fresh vegetables) 69 consumers and sample number 4 (frozen fish, frozen vegetables) 65 consumers. Of all the respondents, 67.3% were women and 32.2% men. One participant did not check the gender box in the questionnaire. The average age of the participants was 46 ± 12.2 years ranging from 22 to 87 years. The questionnaire was formulated in Finnish. In the consumer study, three soups of the four were used, these were numbers 2, 3, and 4 as depicted in Table 1. The sample with only fresh ingredients was excluded.

The study was conducted in a foodservice restaurant that only served a buffet lunch from 10.30 a.m. to 2 p.m., and typically had 300 customers daily. The consumers were contacted at the entrance to restaurants and asked to participate. The actual purpose of the study was not introduced. When asked, customers were informed that the purpose of the study was to collect user experiences of fish soup. The customers were asked to complete a questionnaire after eating. Customers paid the regular fee for the lunch serving, but a free dessert was promised as a reward after completing the form. The fish soup was a normal part of the lunch buffet offering, which consisted of three warm dishes and salad components. The customers participating in the study chose to eat the soup willingly. Only one sample was available at each point of time; this meant that each participant evaluated only one of the three samples. The restaurant served a buffet lunch from 10.30 a.m. to 2 p.m., and data were gathered during that time period. The data were gathered during three test days. During each test day, two different types of soups were served to reduce the effect of the day.

The respondents were asked to evaluate their experience of pleasantness at the appearance, smell, taste, fish texture, and vegetable texture. A balanced 5‐point hedonic scale with descriptors was used for pleasantness (1 = dislike extremely, 2 = dislike moderately, 3 = neither like nor dislike, 4 = like moderately, 5 = like extremely). Customers also had the possibility to freely comment on the quality of the fish soup. After finishing their meal, customers returned the questionnaires to a separate collection point and for the completed form they received a free dessert.

### 2.4 Data analysis

In order to recognize the underlying dimensions behind the fish soup properties, a principal component analysis (PCA) was performed . For the PCA, the descriptors chosen by the in‐house panel were used. The PCA was carried out with Unscrambler X (version 10.3, Camo Software, Oslo, Norway).

The differences between the hedonic score ratings for the fish soups were tested using an analysis of variance (ANOVA) using two a priori planned comparisons. The effect of a single component was tested between the sample with only frozen ingredients (4) and samples with either fresh fish (2) or vegetable component (3). All the tests were considered as significant for *p* < .05. The effect sizes were calculated using Cohen's *d*.

Statistical analysis was also performed to verify the effects of the demographic profiles. All statistical analyses were performed with IBM SPSS Statistics, version 22.

## 3 RESULTS AND DISCUSSION

### 3.1 Sensory characteristics

**Figure 1:**
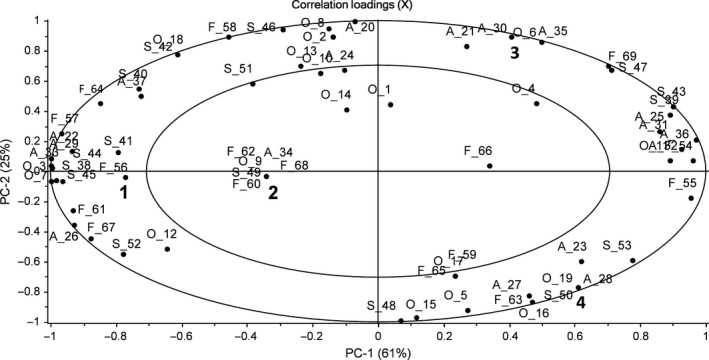
Principal component analysis including all the sensory modalities

A categorical PCA was used to test the data from the check‐all‐that‐apply (CATA) method used with the sensory panel. Results from the categorical PCA for different soup qualities (odor (O), appearance (A), structure (S), and flavor (F)) are presented in Figure 1. The soup samples are presented in Table 1, and the same number codes for each of the soup samples are used in Figure 1. The codes for each descriptor used for different sensory modalities are explained in Table 2. Altogether, 69 descriptors of 72 were used. The three descriptors not used were excluded from Table 2 (slippery (S), stingy (F), and off‐flavor (F)).

**Table 2:** Codes for different descriptors used in the principal component analysis

Odor		Appearance		Structure		Flavor	
1	O_none	20	A_red	38	S_smooth	54	F_none
2	O_mild	21	A_grey	39	S_solid	55	F_mild
3	O_strong	22	A_yellow	40	S_flaky	56	F_strong
4	O_green	23	A_green	41	S_mashed	57	F_sweet
5	O_grass	24	A_pale	42	S_crispy	58	F_salty
6	O_dill	25	A_murky	43	S_dry	59	F_sour
7	O_fishharbour	26	A_bright	44	S_sediment	60	F_bitter
8	O_rawfish	27	A_pasty	45	S_slimy	61	F_umami
9	O_leather	28	A_colorless	46	S_separated	62	F_fishharbour
10	O_sea	29	A_smashed	47	S_hard	63	F_rancid
11	O_seaweed	30	A_solid	48	S_muscular	64	F_fresh
12	O_fatty	31	A_sediment	49	S_sloppy	65	F_artifical
13	O_oil	32	A_even	50	S_even	66	F_veggie
14	O_rancid	33	A_uneven	51	S_uneven	67	F_fat
15	O_veggie	34	A_fiberous	52	S_tattered	68	F_leather
16	O_onion	35	A_strips	53	S_rubbery	69	F_watery
17	O_fusty	36	A_cube				
18	O_sweet	37	A_home				
19	Off‐odor						

As can be seen from Figure 1, the soups differ from each other significantly. This indicates that even with a slight change or replacing one frozen component with a fresh one can cause significant changes to the perceived quality of a dish to a trained person. By reducing the number of variables into two principal components, the effect of the different components in the final sensory characteristics of the fish soup can be interpreted. The first principal component (PC1) explains 61% of the variance. The locations on the figure of the soup sample indicate that the PC1 describes the overall freshness of the soup or in other words the level of processing of the components. Soup (1) containing only fresh ingredients is located on the left‐hand side of the figure, as is the following soup (2) contained fresh fish and a frozen vegetable mix. Based on this, fresh fish contributes more to the overall dish quality than the fresh vegetables. Even if soup (2) is experienced as slightly less fresh, it is still perceived entirely differently to the soup with frozen fish and a fresh vegetable mix. Both the soups with frozen fish are located on the far right of the PC1. The difference between these soups is not very large, but in this case, the fresh vegetable mix induced a fresher perception.

The second principal component (PC2) explains 25% of the total variance. The interpretation of PC2 is slightly more demanding than PC1. Both soups with fresh fish (1 and 2) are located very close to the *x*‐axis and are very neutral in comparison with the soups with frozen fish. These soups differ from each other significantly and are located at the opposite end of the axis. Soup with a fresh vegetable mix (3) received a strong positive loading on PC2, while soup with only frozen ingredients had strong negative loadings. Based on this, PC2 describes the freshness of the vegetable mix only when frozen fish is used. The fresh fish is a much more dominant factor as regard perceived sensory characteristics and therefore the freshness of the vegetables cannot be recognized in this case.

The CATA method was a valuable tool for differentiating the samples. The results show that different combinations of the components produce a variance in the sensory properties perceived by the sensory panel. As can be seen from the Figure 1, fresh components produce a strong and fatty flavor (fat, umami, strong, and sweet), whereas the closest descriptors for the flavor of the soup with only frozen ingredients were sour, rancid, and artificial. Similar descriptors are close to the properties attached to a cooked fish with fresh‐like qualities (meaty flavors with slight sweetness) while for less fresh, the typical properties are relating to bitter, sour, and off‐flavors (Alasalvar et al., 2001). If the characteristics are not typical of a fresh product, it may be considered as artificial or processed, thus inducing negative reactions towards the product (Ares et al., 2016). It may be that stronger and more intense flavors improve, for example, food palatability and acceptance as indicated in the study by Schiffman (2000).

The appearance of the soup with fresh components was described as uneven, bright, yellow, and smashed, and the structure, for example, smooth and tattered. The soup with only frozen ingredients, on the other hand, was described as colorless, green, and pasty, with the structure described as even and rubbery. Both the appearance and structure may have an impact on the perceived product properties in context of being consumed. For instance, a dish containing uncharacteristic colors (Spence, 2015a) or textures (Szczesniak, 2002) may cause rejection. The closest descriptors for odor were strong, fat, and fish harbor when only fresh ingredients were used. When the soup was made using frozen fish and vegetables, the closest descriptors for odor were off‐odor and onion. Odor may have a significant impact as to whether a dish is rejected or not. People tend to respond to unpleasant food odors more rapidly and accurately than to pleasant ones (Boesveldt, Frasnelli, Gordon, & Lundström, 2010). This may infer that if unpleasant odors are present in the fish soup, it may lead to negative evaluations and to possible rejection as the desired qualities or odors may be overridden.

### 3.2 Consumer study

In the consumer study, the soups were perceived differently in a regular consumption context. The average hedonic scores for the fish soup samples and the results from planned comparisons (*p*‐values) with calculated effect sizes (Cohen's *d*) are presented in Table 3. The soup with fresh fish (2) received the highest evaluations for appearance, taste, and fish texture with statistically significant differences comparing to sample 4. The soup with fresh vegetable mix (3) was evaluated to be significantly more pleasant for taste and fish texture than the soup with frozen ingredients (4). The effect sizes vary from small to large where the greatest effects can be seen for taste and fish texture. For vegetable texture and appearance, the effect sizes can be considered being closer to medium, which is already visible to the naked eye, according to the conventional definitions proposed by (Cohen, 1988) (small = 0.2, medium = 0.5, large = 0.8).

**Table 3:** The average hedonic scores for the fish soup samples including the results from planned comparisons (2 vs. 4 and 3 vs. 4) with effect sizes. (STD/Cohen's *d*) [*n*]

Sample	Description	Appearance	Smell	Taste	Fish texture	Vegetable texture
2	Fresh fish, frozen vegetables	3.99^b^ (0.57/0.44) [71]	3.79^a^ (0.66/0.16) [70]	4.24^b^ (0.55/0.82) [70]	4.17^b^ (0.64/0.63) [70]	3.97^a^ (0.61/0.26) [71]
3	Frozen fish, fresh vegetables	3.88^a^ (0.74/0.25) [69]	3.81^a^ (0.65/0.20) [69]	3.98^b^ (0.63/0.37) [69}	3.91^a^ (0.84/0.27) [68]	4.03^b^ (0.62/0.35) [68]
4	Frozen fish, frozen vegetables	3.71^a^ (0.70) [64]	3.68^a^ (0.69) [64]	3.73^a^ (0.70) [63]	3.67^a^ (0.96) [63]	3.81^a^ (0.64) [63]

Values followed by different letters are significantly different (*p* < .05).

The evaluated taste was more pleasant for both the soups with fresh ingredients and the differences were statistically significant. Fresh fish seemed to have the greatest effect on the pleasantness of the soup. By replacing frozen fish with fresh, there is a possibility to increase the perceived sensory quality of fish soup significantly. Even though the differences were significant, the absolute differences were rather small. This may be due to the fact that if the quality of the fish has not changed dramatically during storage, cooking may mask some of the undesirable properties; this may diminish the differences perceived by the consumers (Alasalvar et al., 2001). Interestingly, the difference between the soups with frozen fish and varying vegetable component is also statistically significant, but not as great as the one with fresh fish. Different components seem to be meaningful, but unequally balanced with regard to certain sensory properties. Previous studies show that a single meal component also affects how other components are evaluated (Jimenez et al., 2015; Nestrud, Ennis, & Lawless, 2012). However, in this case, the interest was not the effect of a combination of components, but the effect of the component quality on overall quality. Thus, fresh fish has the ability to enhance the taste of the soup, even with the frozen vegetables. This indicates that in the case of fish soup, the fish component carries more weight than the vegetable component.

For the soup with fresh fish (2), the texture of the fish received the highest rating and the difference was significant when compared to the soup made purely from frozen ingredients (4). The texture of the vegetables showed a similar type of phenomenon. The pleasantness of the vegetable texture is at the highest level with fresh vegetables, and the difference was significant when comparing to the sample 4. The term textural contrast is used when, for example, a dish contains a variety of different textures (Szczesniak, 2002). According to Szczesniak (2002), the most pleasant texture combinations are the ones with the large differences (e.g., crisp/creamy). When the textures of the fish and vegetables are evaluated as a part of a dish, the combination of textures may be more relevant than the texture of an individual component. Because of this, the fresh vegetables may also have an effect on the evaluated fish texture and diminish the effect of a single component in the consumer study. In addition, other modalities may have an impact, for example, a study by Michon, Sullivan, Sheehan, Delahunty, and Kerry (2010). showed that the flavor intensity of soup has the potential to influence the perceived appearance and as well as the texture. It may be due to this that the perceived appearance of the soup with fresh fish was evaluated being more pleasant than the ones with frozen fish.

The soup smell was the only quality with no statistically significant differences. When fish soup is made using similar ingredients with varying quality, the properties of the finished products may be very close to one and other. The consumers may not be sensitive enough to detect the differences between the pleasantness of all the evaluated properties and the differences remain insignificant. A study by Michon et al. (2010) using a series of soup samples showed that even though the sensory properties differed from each other, no difference was shown between the consumer liking of the products. The role of smell in the experienced quality may be largely dependent on the food in question (Spence, 2015b). When the samples were not differentiated based on the smell, other modalities may be more dominant (Fenko, Schifferstein, Huang, & Hekkert, 2009). It may be that in the soup samples, the combinations of fresh and frozen components produced a mixture of pleasant and unpleasant odors. For example, Grabenhorst, Rolls, Margot, da Silva, and Velazco (2007) showed that when a mixture of pleasant and unpleasant odors are present, the olfactory system of the brain can simultaneously produce negative and positive hedonic values affecting the decision‐making system.

### 3.3 General discussion

Unprocessed components induce sensory characteristics in the final product, which can be seen as desirable. There is previous evidence that certain meal or dish compositions are more appealing than others (Aaslyng & Frøst, 2010; Nestrud et al., 2012). The results from the sensory panel and consumer study indicate that different food components are unequally balanced: The unprocessed fish produced more fresh‐like or desirable soup qualities than the fresh vegetable mix. This is in line with previous research that the main component or meal center dominates the acceptance effect (Hedderley & Meiselman, 1995; Turner & Collison, 1988). It will require further research, to assess whether the same kind of procedures can be applied to other food products. By introducing this type of operational practice, the sensory quality of complete dishes could be optimized using certain strategically selected components to enhance the final quality. Using this type of method, the quality, for example professional kitchens and manufacturing processes, could be enhanced without changing the raw material mix entirely. Companies serving modular dishes take a considered risk by varying the quality of different components. When using inferior component as a part of the dish, the decrease in the level of quality could be estimated in advance.

It is common practice in product development to study the effects of different components and spices on the sensory properties of a food product. This same kind of procedure could also be applicable in complete dishes. By mixing different food components and optimizing the overall quality, even the perceived quality of inferior components can be increased. When considering a complete meal or dish, during cooking, the aromas and taste inducing components interact with each other (Meinert, Frøst, Bejerholm, & Aaslyng, 2011). Prior studies have shown that by recognizing and optimizing the combinations of different components, the dish quality can be increased (Meinert et al., 2011; Nestrud et al., 2012). In this study, the main purpose was to optimize the sensory quality or to extract the best qualities of a single dish component. By developing a modular dish concept from a customer's perspective, the overall quality could be increased without compromising efficiency. This concept still needs further development, but it can be a useful tool to optimize large‐scale processes.

In this study, the products were configured from two components with two possible choices. Each of the combinations produced a very different customer experience. Even with a very narrow selection of components, four different versions of the same product can be made. When starting to optimize the sensory properties and operational efficiency, it is possible to utilize modularization as a tool. This type of modularity is aimed mainly at foodservice restaurants or food manufacturers serving a large number of customers with a limited variety of moderately priced dishes. Previous research has shown evidence that manufacturing companies (e.g., electronic equipment) can utilize modular product design effectively to facilitate product variety without additional cost (Piran, Lacerda, Antunes, Viero, & Dresch, 2016; Shaik, Rao, & Rao, 2014). A review by Vickery, Koufteros, Dröge, and Calantone (2016) showed evidence that in the context of traditional manufacturing industries (machinery, computers, and transportation equipment), adding modularity to the product design can have a significant effect on new product performance and accelerate the development process. As there are only a very few studies on mass customization in the food industry (Adler‐Nissen et al., 2013; Olsen & Aaslyng, 2007), the applicability of modularity to a complete dish on a larger scale and the effect on perceived quality needs to be studied further. With a modular design, the company serving the dish can add flexibility to the production processes and raw material choices. In addition, by adding interchangeability into the matrix, some components could be replaced. This would enable the usage of seasonal vegetables or other products that are not continuously available, and thus to manufacture the best possible quality with the available resources, as well as optimize the balance between product quality and price.

The generalizability of these results is subject to certain limitations. The most important limitation lies in the fact that in the test case, only one type of product was used. The applicability to other raw materials and products will require further testing. In addition, the study was conducted in only one country with an individual food culture, and therefore, a similar effect in other countries and cultures cannot be verified.

## 4 CONCLUSIONS

The present study showed that modularity, or in the food context, a dish separated into components can be applied to the foodservice industry. In this case, the tested product was fish soup and the freshness of the fish and the vegetable mix components were varied. The results of this study indicate that each component can have an effect on the dish quality. The CATA method was a valuable tool for differentiating the samples. The results show that different combinations of the components produce a variance in the sensory properties perceived by the sensory panel. In the consumer studies, soup prepared only using preprocessed components received the lowest hedonic scores for all the evaluated properties (appearance, smell, taste, fish texture, and vegetable textures) with significant differences expect in the case of smell. Replacing a preprocessed component with a fresh one increased the perceived pleasantness of the fish soup. The fish as the main dish component had the largest effect on the quality. The fresh vegetable component also had an effect on the quality, but the difference was not as great as with the fish component. The significance of the dish center appears to be more prominent than the vegetable or carbohydrate component. The results from this study indicate that the effect of freshness can also be perceived in the cooked product.

## CONFLICT OF INTEREST

None declared.
